# Dealloying fabrication of hierarchical porous Nickel–Iron foams for efficient oxygen evolution reaction

**DOI:** 10.3389/fchem.2022.1047398

**Published:** 2022-11-03

**Authors:** Tingting Zhou, Zilong Liu, Bei Yang, Zhen Cao, Zaiyong Jiang, Weiran Cui, Kaili Wang, Lei Yu, Jitao Lu, Ling Zhang

**Affiliations:** ^1^ College of Chemical Engineering and Environmental Chemistry, Weifang University, Weifang, China; ^2^ School of Science, Harbin Institute of Technology, Shenzhen, China

**Keywords:** electrocataiysis, dealloyed, hierarchal structure, nickel-iron (NiFe), oxygen evolution reduction

## Abstract

Designing and preparing highly active oxygen evolution reaction (OER) electrodes are essential for improving the overall efficiency of water splitting. Increasing the number of active sites is the simplest way to enhance OER performance. Herein, we present a dealloy-etched Ni–Fe foam with a hierarchical nanoporous structure as integrated electrodes with excellent performance for OER. Using the dealloying method on the Ni–Fe foam framework, a nanoporous structure is produced, which is named nanoporous Ni–Fe@Ni–Fe foam (NP-NF@NFF). Because of the peculiarities of the dealloying method, the NP-NF@NFF produced contains oxygen vacancies and heterojunctions. As a result, NP-NF@NFF electrode outperforms state-of-the-art noble metal catalysts with an extremely low overpotential of 210 and 285 mV at current densities of 10 and 100 mA cm^−2^, respectively. Additionally, the NP-NF@NFF electrode shows a 60-h stability period. Therefore, NP-NF@NFF provides new insights into the investigation of high-performance transition metal foam electrodes with effective active sites for efficient oxygen evolution at high current densities.

## 1 Introduction

Electrochemical water splitting is considered one of the most promising environmentally friendly methods for producing hydrogen (H_2_) because of its characteristics as a nonpolluting and abundant source (Chen et al., 2021; Jin et al., 2022). Moreover, it could convert excess power into chemical energy from renewable sources and help in smoothing out minor fluctuations in power, thus improving the efficiency of the power grid (Lewis and Nocera, 2006; Cook et al., 2010). However, the inferior energy conversion efficiency of hydrogen production *via* electrochemical water splitting is highly restricted by the anodic oxygen evolution reaction (OER) due to the intrinsically sluggish reaction kinetics (Liu et al., 2021; Tang et al., 2022; Yu et al., 2022). Consequently, considerable effort has been devoted to exploring high-performance electrocatalysts capable of accelerating electron transfer and reducing or even breaking the kinetic bottleneck. Noble metals (e.g., Ru and Ir) and their oxides (e.g., RuO_2_ and IrO_2_) are the most well-known electrocatalysts for OER. However, their large-scale application has been limited by their high cost, scarcity and weak alkaline stability (Wang et al., 2022a). Consequently, it is highly desirable to develop durable, low-cost electrocatalysts for OER with high efficiency.

In recent years, researchers have extensively examined the oxygen evolution activities of a large number of transition metal catalysts, and their application potential has been demonstrated (Kou et al., 2021; Chatenet et al., 2022; Sen et al., 2022). In general, preparing powdered catalysts into electrodes usually requires the use of binders, which are susceptible to problems such as active site coverage, conductivity hindrance and catalyst peeling (Li et al., 2022). In view of the aforementioned problems, many researchers choose to directly grow the catalyst on foam metals with high conductivity and millimetre-sized pores, such as Fe–Zn MOFs/Ni foam, (Wang et al., 2022b), Ni_3_S_2_@CeO_2_/Ni foam, (Wu et al., 2021), a-Ni–Fe nanosheet/Fe foam, (Yang et al., 2019), Ni–Fe-MOFs/Ni–Fe foam (NFF), (Zhao et al., 2021), and Co–Fe LDH/Cu foam (Zhou et al., 2017). Additionally, the integrated electrode can not only shield the interference of the binders but also improve the electrolyte flux to aid in bubble overflow.

The OER performance of catalysts is generally determined by two factors: intrinsic activity and the number of active sites (Seh et al., 2017). Therefore, by increasing the surface area of the substrate, the performance of the electrode can be greatly enhanced. Additionally, when the specific surface area of the substrate increases, a large number of active sites exposed by the substrate itself can play a significant role in OER, achieving an effect of dual advantages (Wu et al., 2019). Dealloying is an easily achievable strategy in many nanoporous fabrication techniques, and it has been widely used in the production of electrocatalysts (Chen et al., 2018; Fu et al., 2022). However, there are no reports on the modification of metal foam substrates. In this study, the dealloying technique is applied to create nanoporous structures on commercial NFFs. Because of the increased surface area and heterojunction formation caused by the dealloying technique, the nanoporous Ni–Fe@NFF (NP-NF@NFF) resulting from the dealloying strategy enables the substrate to show excellent OER performance. Benefiting from significantly exposed active sites and enhanced electrical conductivity, the obtained NP-NF@NFF electrode showed greater ultrahigh current density and prolonged stability towards OER than NFF and commercial IrOx.

## 2 Experimental section

### 2.1 Chemical reagents

Zinc vitriol (ZnSO_4_·7H_2_O), sodium hydroxide (NaOH), potassium hydroxide (KOH), acetone [(CH_3_)_2_CO], ethanol (CH_3_CH_2_OH), saccharin (C_7_H_5_O_3_NS), citric acid (C_6_H_8_O_7_) and sodium dodecyl sulfate (SDS, C_12_H_25_SO_4_Na) were supplied by Sinopharm Chemical Reagent Co. Ltd., Shanghai, China. The commercial IrOx was supplied by Alfa Aesar, Ward Hill, MA, United States. Nafion (5 wt%) was supplied by Shanghai Branch, Du Pont China Holding Co., Ltd. Shanghai, China. The NFF was supplied by Kunshan Tengerhui Electronic Technology Co., Ltd., Shanghai, China.

### 2.2 Synthesis of NP-NF@NFF

The as-prepared NFF was used as the substrate to plate Zn. The plating solution was prepared by dissolving 2.87 g ZnSO_4_·7H_2_O, 4.0 g H_3_BO_3_, 0.5 g saccharin and 0.1 g SDS citric acid in 100 ml of ultrapure water under continuous stirring. The pH was adjusted to 4.0 by adding diluted sulfuric acid drop by drop. The electrodeposition was performed in a two-electrode cell, with the FeNi3 foam and Zn plate used as working and counter electrodes, respectively. Cathodic electrodeposition was performed at a current density of 50 mA cm^−2^ for 10 min at a temperature of 80°C. The obtained NFF coated with Zn (Zn@NFF) was rinsed with ultrapure water several times and dried under a vacuum. Then, the sample was calcined in argon at a temperature of 500°C for 4 h with a heating rate of 5°C min^−1^. The dealloying procedure was performed in 100 ml of 1 M citric acid for 15 min at 30°C. The NP-NF@NFF was then obtained. Finally, thorough washing with ultrapure water many times and drying under a vacuum were carried out.

### 2.3 Material characterisation

The crystal structures of the samples were obtained using a SmartLab diffractometer equipped with Cu Kα radiation. The morphology and microstructure of the samples were acquired using scanning electron microscopy (SEM, ZEISS MERLIN) and transmission electron microscopy (TEM, FEI Tecnai G2 T20). The measurements for oxygen vacancy (Ov) were characterised using electron spin resonance (ESR) (Bruker EMXplus). The surface properties were collected using X-ray photoelectron spectroscopy (XPS, PHI 5000 VersaProbe).

### 2.4 Electrochemical measurements

The electrochemical experiments were performed using a typical three-electrode cell at 25°C utilising a CHI 760E electrochemical workstation (CH Instruments, Inc. Shanghai). As an electrolyte, 1-M O2-saturated KOH (pH = ∼14.0) was utilised. A piece of NP-NF@NFF (1 × 1 cm^2^) was applied as the working electrode, and a graphite rod and an Hg/HgO electrode (1 M KOH) were used as the counter and reference electrodes, respectively. The commercial catalyst electrode was prepared as follows: 4.0 mg of electrocatalyst powder was dispersed in 420 µL of ethanol, 500 µL of water and 80 μL of Nafion solution and then sonicated for 30 min. On clean carbon paper (1 × 1 cm^2^), 50 μL of ink was dropped and dried at 60°C. All the scanning cyclic voltammogram (CV) and linear sweep voltammetry (LSV) measurements were performed at a sweep rate of 5 mV s^−1^ with an iR compensation. Stability testing was performed using the chronoamperometric method. The electrochemical impedance spectroscopy (EIS) measurements were performed at a given potential of 1.50 V with an amplitude of 5 mV over a frequency range of 10−1–105 Hz. Electrochemical surface areas (ECSAs) are calculated on the basis of the following formula: ECSAs = Cdl/Cs. Here the values of the double-layer capacitance (Cdl) were determined by scanning CV curves at various scan rates in the range of 0.90–0.95 V vs. reversible hydrogen electrode (RHE), whereas Cs is usually adopted as 4 μF cm^−2^ for Ni-based catalysts. (McCrory et al., 2013). The measured potentials were calibrated in terms of RHE.

## 3 Results and discussion

**FIGURE 1:**
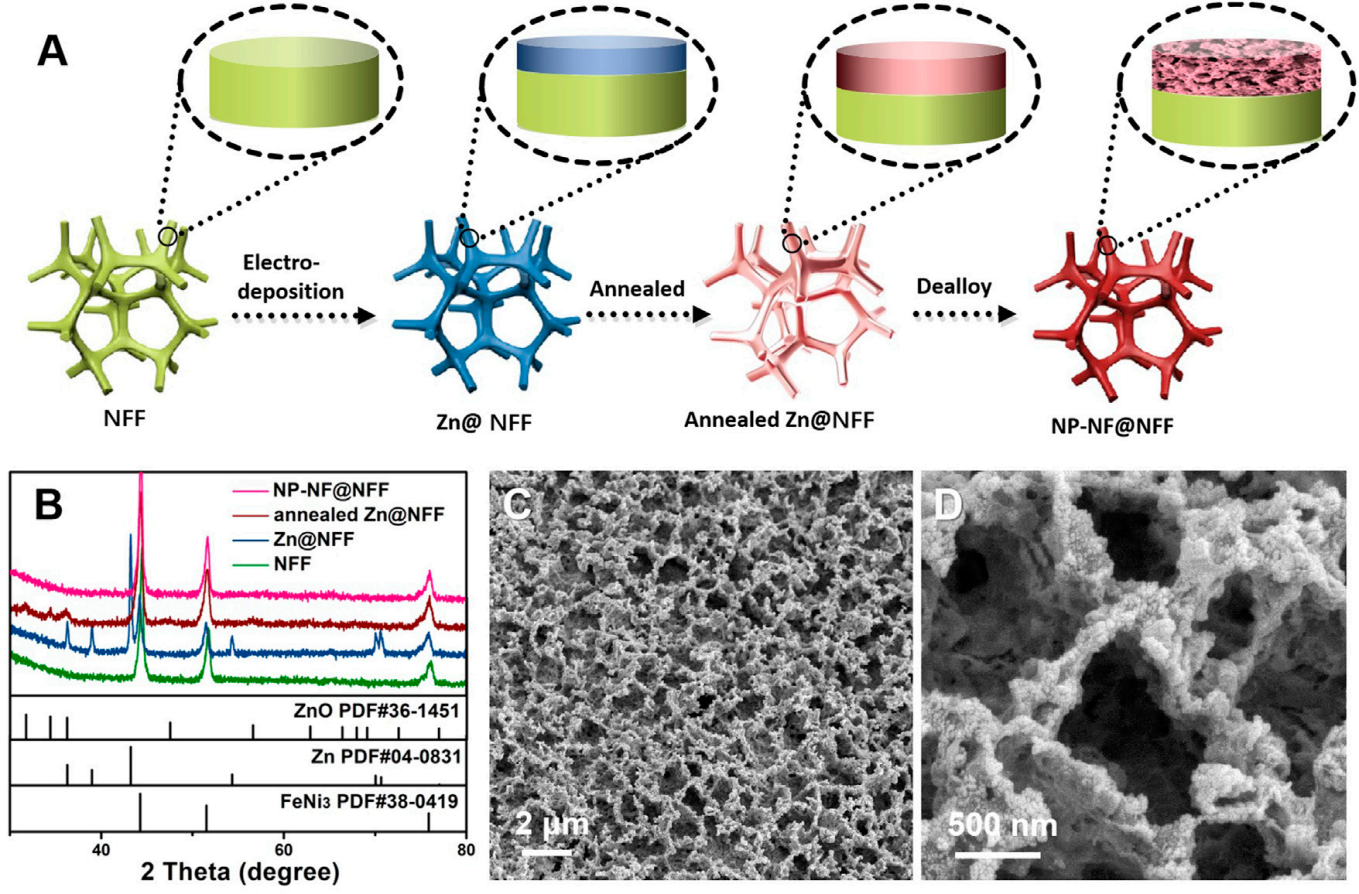
**(A)** Illustration of the electroplating–annealing–dealloying fabrication process of NP-NF@NFF; **(B)** XRD patterns of NFF, Zn@NFF, annealed Zn@NFF and NP-NF@NFF; and **(C)** and **(D)** SEM images of NP-NF@NFF.

Active components of an alloy were referentially dissolved, whereas the stable components were retained, resulting in the formation of a nanoporous state based on the dealloying principle. Before dealloying, an active metal that can be corroded in the material system is required. Here, we have chosen Zn as the active component and Ni and Fe as the stable components to create a nanoporous structure. In brief, we developed an electroplating–annealing–dealloying (EAD) method to fabricate nanoporous Ni–Fe with a hierarchical nanoporous structure integrated onto NFF. Figure 1A shows a schematic illustration of the EAD fabrication process. The Zn coating was prepared on the surface of the NFF substrate using the electrodeposition method. Subsequently, an annealing process was used to promote the formation of a precursor alloy on the surface of the NFF substrate. Then, mildly corrosive citric acid was chosen as the dealloying solution to selectively remove Zn and produce a 3D nanoporous structure. X-ray diffraction (XRD) was used to reveal the structural changes that occurred during the EAD process (Figure 1). The NFF shows diffraction peaks at 44.3°, 51.5°, and 75.9°, corresponding to the (111), (200) and (220) planes of FeNi3 (JCPDS No. 38-0419), respectively. These three peaks appeared in all EAD samples, and their width and strength remained unchanged, showing that the NFF skeleton remains unchanged. Zn@NFF shows additional diffraction peaks at 36.2°, 39.0°, 43.2°, 54.3°, 70.1°, and 77.0°, corresponding to the (002), (100), (101), (102), (103) and (004) planes of Zn (JCPDS No. 04-0831), respectively. After annealing, the diffraction peaks corresponding to Zn vanished and were replaced with weak diffraction peaks corresponding to ZnO (JCPDS No. 04-0831). At high temperatures, most of the Zn diffuses into the Ni–Fe alloy lattice, whereas a small amount of Zn is oxidised to ZnO. After dealloying, the NP-NF@NFF end product showed similar XRD patterns to the NFF. Generally, dealloying leads to partial oxidation of transition metals. However, the apparent diffraction peaks corresponding to hydroxide or oxide were not observed in the XRD data of NP-NF@NFF. This may be due to the low content and low crystallinity of hydroxide or oxide loaded on the surface of NFF. Significantly, the XRD pattern of NP-NF@NFF no longer gave obvious the diffraction peaks of Zn and Zn compound, but a small amount of residue may still exist according to the mechanism of dealloying. Some studies hold that a small amount of residual active metal will not affect the performance of OER. (Ponce et al., 1999; Dong et al., 2018), but some studies also point out that the residue is harmful to OER (Detsi et al., 2016) No matter whether the residue is harmful to OER, it does not affect its ability to successfully construct nanopores. A large number of active sites provided by nanoporous structures must be conducive to catalysis.

Figures 1C,D; Supplementary Figures S1,S2 show SEM images of the surface morphology of pristine NFF and NP-NF@NFF. The morphology of pristine NFF is very flat, and no significant nano-structure can be observed, as shown in Supplementary Figure S1. Morphology transformation has occurred after dealloying (Supplementary Figures S1,S2). From the SEM images of NP-NF@NFF, the bicontinuous interpenetrating porous 3D network can be observed, which is constructed with interconnected macropores with a diameter of 420 nm (Supplementary Figure S3). Additionally, it is worth noting that in the high-magnification SEM image (Figure 1D), it can be observed that there are nanopores in the generated macroporous framework.

**FIGURE 2:**
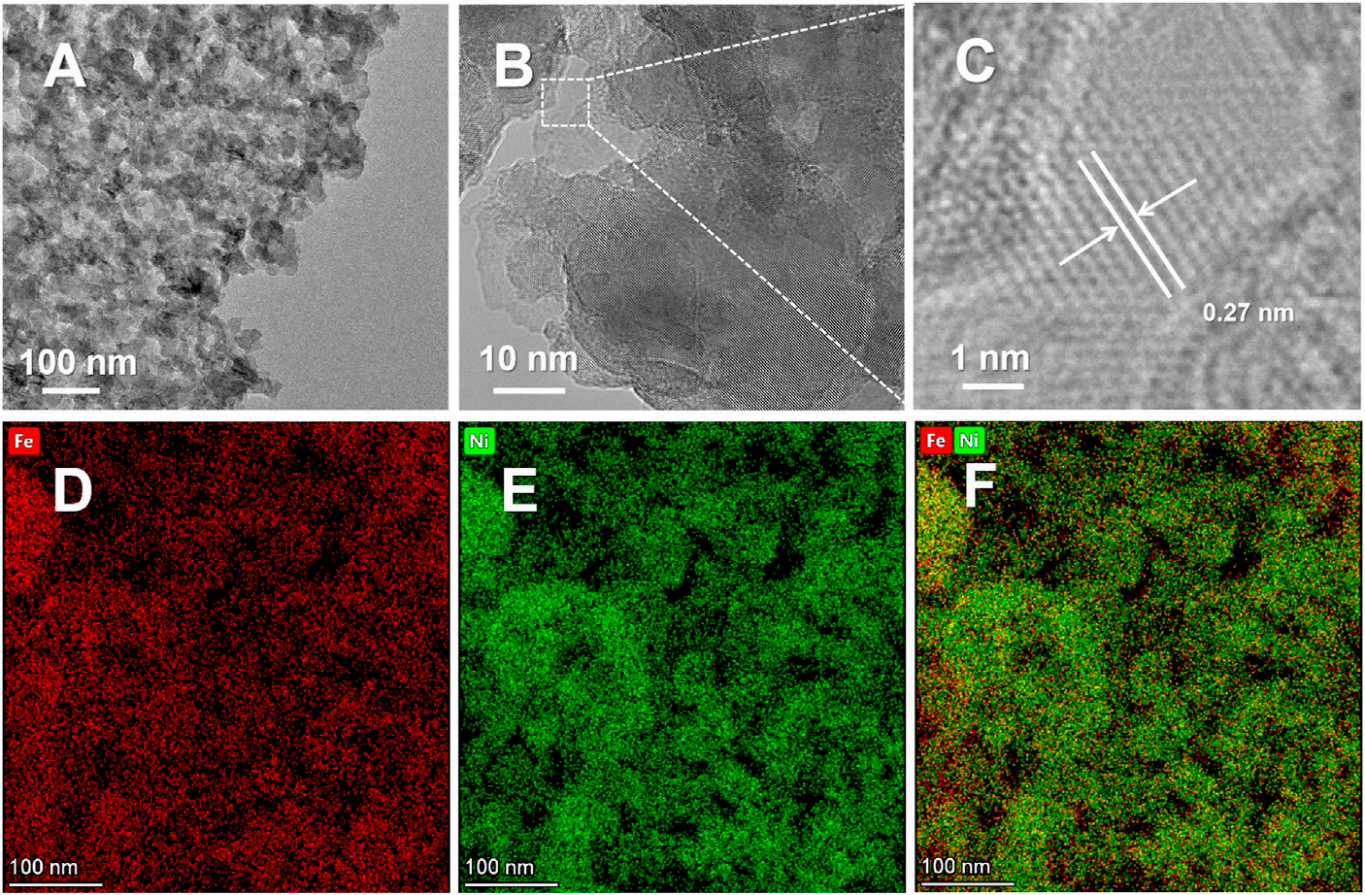
**(A)** Low-magnification TEM image, **(B)** high-magnification TEM image, **(C)** high-resolution TEM and **(D–F)** the corresponding EDS elemental mapping of NP-NF@NFF.

The nanoporous structure of NP-NF@NFF was further examined using TEM. TEM images in Figures 2A,B show the secondary nanoporous structure formed by bicontinuously connected nanoparticles with diameters of 30–50 nm and the resulting pores with diameters of 20–30 nm. It is worth noting that hierarchical morphology not only improves the external surface area but also facilitates mass transfer. The macropores were responsible for mass transfer, but the mesopores could expose more active sites (Zhen et al., 2019). The high-resolution TEM image in Figure 2C shows additional details, showing that NP-NF@NFF has lattices with a spacing of 0.27 nm, correspond to the (100) plane of the Ni(OH)_2_. Obviously, these hydroxides loaded on the surface of NFF was not observed by XRD due to the low content and crystallinity. Additionally, the distribution of Ni and Fe elements is uniform (Figures 2D–F), showing that element segregation will not result from the dealloying method of pore formation. The results show that the Ni–Fe-based a hierarchical nanoporous structure was successfully fabricated on the NFF surface, but the specific chemical state of the surface needs to be confirmed.

**FIGURE 3:**
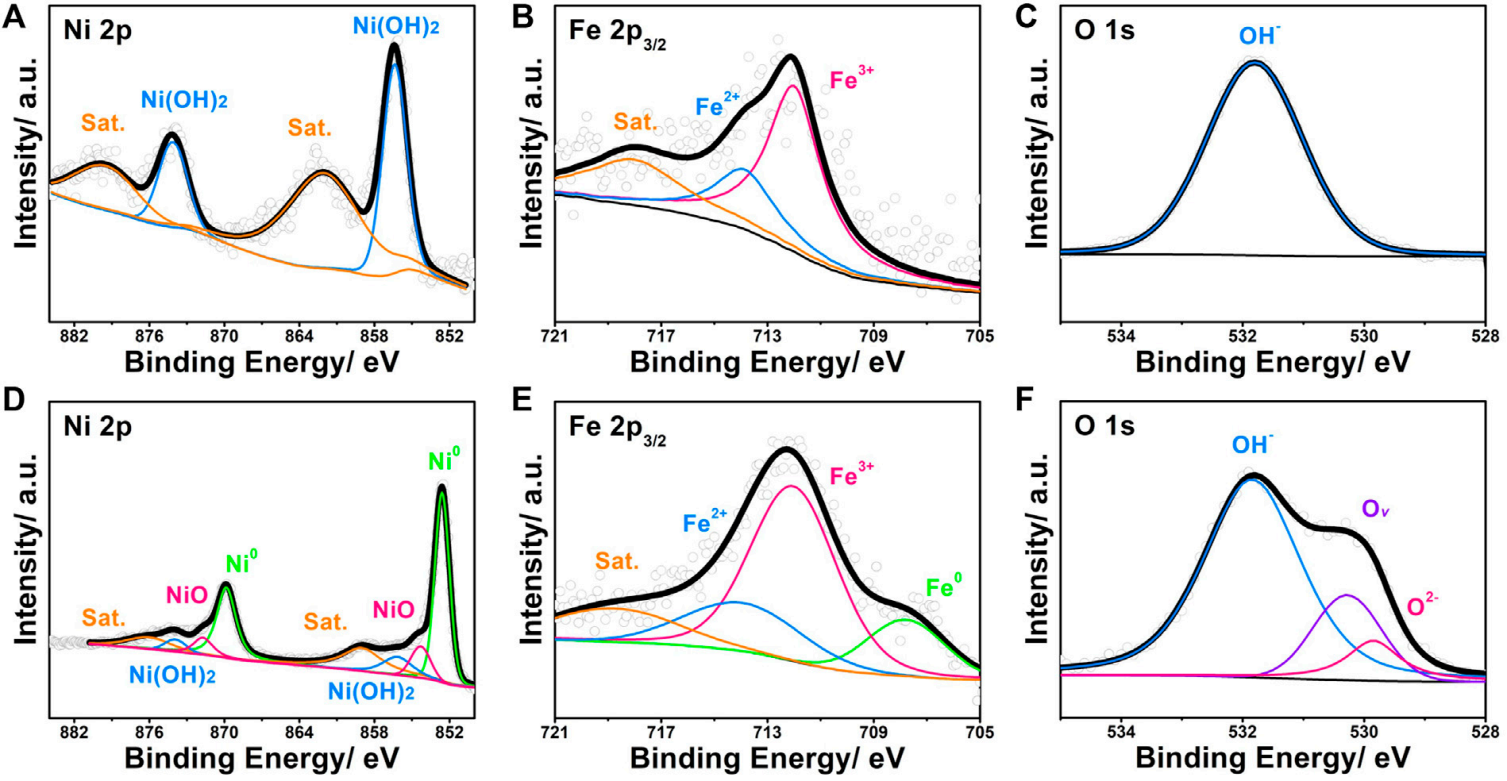
High-resolution **(A)** Ni 2p XPS spectra, **(B)** Fe 2p3/2 XPS spectra and **(C)** O 1s XPS spectra of NP-NF@NFF. High-resolution **(D)** Ni 2p XPS spectra, **(E)** Fe 2p3/2 XPS spectra and **(F)** O 1s XPS spectra of NP-NF@NFF after etching.

Concerning the surface state of NP-NF@NFF, we utilise XPS for additional characterisation. Additionally, the internal chemical state of NP-NF@NFF was measured using the argon ion etching method attached to the same XPS equipment. The specific analysis of the interaction between hydroxide and metal usually depends on the results of XPS etching. The Fe and Ni 2p XPS spectra of the surface (Figures 3A–C) and internal (Figures 3D–F) with the etching time of 5 min are quite different. For the surface of NP-NF@NFF, the splitting peaks at 856.4, 862.6, 874.1 and 879.5 eV were derived from Ni 2p, as shown in Figure 3A. The splitting peaks at 856.4 and 874.1 eV correspond to 2p3/2 and 2p1/2 of Ni(OH)2, respectively (Wang et al., 2017; Xie et al., 2018). The remaining peaks correspond to their respective satellite peaks. As shown in Figure 3B, the splitting peaks of 712.0, 713.9 and 718.0 eV were derived from Fe 2p3/2. The peak at 712.0 eV corresponds to Fe^3+^, whereas the peak at 713.9 eV should correspond to Fe^2+^ (Nai et al., 2013). As shown in Figure 3C, the high-resolution spectra of O1s can only be fited with peak at binding energy of 531.8 eV, corresponding to OH^−^ (Nai et al., 2013). Thus, it is possible to confirm that the outermost layer of NP-NF@NFF should consist of Ni–Fe hydroxide. After 5 min of argon ion etching, the XPS spectra of NP-NF@NFF corresponding to nickel oxides (854.3 and 871.7 eV), (Dong et al., 2018; Cao et al., 2020), metallic nickel (852.6 and 869.8 eV) and metallic iron (707.8 eV), as shown in the comparison between Figures 3D,E (Jothi et al., 2020). The appearance of oxides is basically similar to the previously reported dealloying phenomenon, which comes from the inevitable oxidation that occurs during the corrosion process. The metallic Ni and Fe should be attributed to the skeleton inside the alloy foam. Additionally, it is worth noting that Figure 3E shows an unusual change in the condition of oxygen. Oxides (529.8 eV) and Ov (530.3 eV) appear (Nai et al., 2013; Lv et al., 2021; Zhou et al., 2022). After the active metal ions have been dissolved during the dealloying process, the oxidation conditions may not be able to reach the interior of the material. Moreover, the ESR spectrum (Supplementary Figure S4) shows a significant symmetrical signal at g = 2.001, whereas a relatively weak signal is observed in NFF, which is consistent with the XPS results. Significantly, the Ov can be used as electron donors to optimise the electronic structure (Lv et al., 2021). The compounds on the surface and the metallic inside can form an interface structure. Heterojunction structure often occurs at the interface of transition metal porous materials obtained by dealloying, (Detsi et al., 2016; Dong et al., 2018; Cao et al., 2020; Fang et al., 2020), which is conductive to charge separation (Jiang et al., 2018). Additionally, compared with regular pores with positive curvature constructed by ordinary nanopore forming technologies, the surface state of 3D bicontinuous pores obtained by dealloying strategy is more complicated. Which usually have the characteristics of negative, positive and saddle-point curvature (Sang et al., 2022) (Fujita et al., 2008). Therefore, the alloy part of NP-NF@NFF may possess these properties. The unique pore structure will form a high proportion of defects, which stems from their need to geometrically adapt to the complex topological structure of the pore. (Rösner et al., 2007; Liu et al., 2016). The coordination unsaturated defect sites can promote the formation of electrophilic oxygen ligands. Which is conducive to reducing the kinetic barriers to the formation of O-O in the oxygen evolution progress, thus improving the OER activity. (Nong et al., 2018). Meanwhile, the synergistic effect of Ov at the interface is more favourable to electron transfer. Therefore, by dealloying NFF not only provide large specific surface area but also create heterojunctions to reducing the kinetic barriers of OER. NP-NF@NFF obtained by dealloying pore forming strategy may bring unexpected OER performance changes.

**FIGURE 4:**
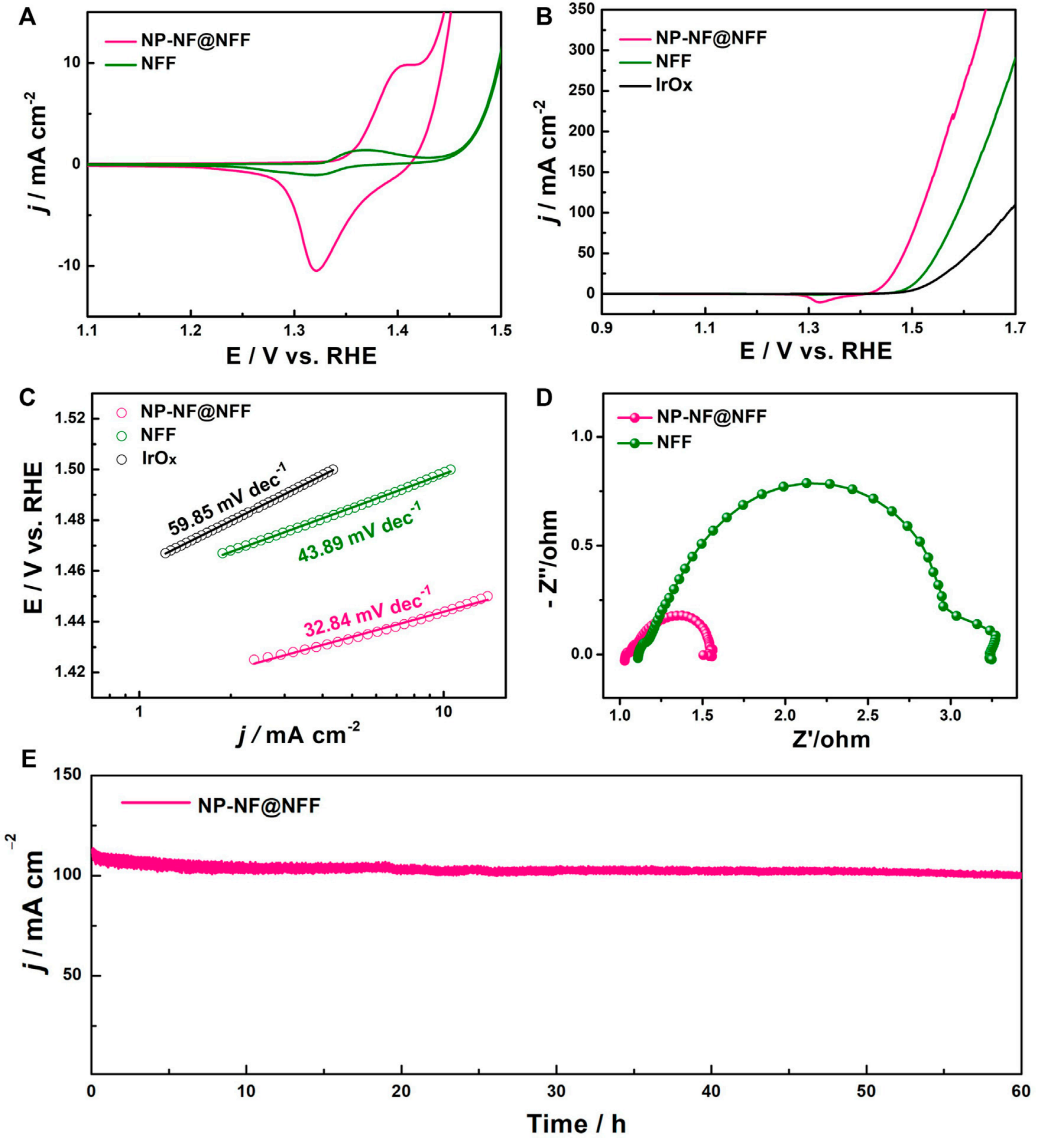
**(A)** Cyclic voltammetry curves, **(B)** linear sweep voltammetry curves and **(C)** corresponding Tafel slopes of NP-NF@NFF, NFF and IrOx; **(D)** Nyquist plots of NP-NF@NFF and NFF; and **(E)** chronopotentiometry curve at 1.53 V vs. RHE for NP-NF@NFF.

The NP-NF@NFF can be directly used as catalytically active electrodes for OER in an alkaline solution, and the NFF and state-of-the-art IrOx were tested as reference. Figure 4A compares the CV obtained for NP-NF@NFF and NFF. According to a previous study, (Wei et al., 2015), the redox feature at 1.4 V can be attributed to the Ni^2+^/Ni^3+^ redox transition. The number of Ni sites that are electrochemically active can be proportional to the surface of the oxidation peak (Corrigan and Bendert, 1989). The integrated charge in the first anodic peak of NP-NF@NFF is almost twice that of NFF, showing that the proportion of the active surface area is similar. This result is consistent with the ECSAs calculated from the CV curves in Supplementary Figure S5. The OER electrocatalytic activity was investigated using LSV curves (Figure 4B). The NP-NF@NFF shows a lower onset overpotential of 185 mV and a significantly faster increase in current density compared with NFF (225 mV) and IrOx (235 mV). The NP-NF@NFF produced a current density of 10 and 100 mA cm^−2^ at an overpotential of 210 and 285 mV, respectively, which was lower than that of NFF and IrOx. And beyond that, the performance of NP-NF@NFF (Table 1) can be compared with the recent excellent Ni–Fe-based electrocatalysts (Supplementary Table S3). These results clearly confirm the value-added transformation of purchased NFF into a highly efficient electrode to provide more active sites for OER using a dealloyed strategy. Additionally, both NP-NF@NFF and NFF possess an electrocatalytic activity that even surpasses that of the commercial IrOx electrocatalyst, which is presumably due to the appropriate inherent advantages of Ni–Fe OER catalysts (Smith et al., 2013) and the excellent electronic conductivity of the alloy substrate. The corresponding Tafel plots of NP-NF@NFF, NFF, and IrOx are shown in Figure 4C; Table 1. The Tafel slope of NP-NF@NFF is determined to be 32.84 mV dec^−1^, which is much less than that of NFF (43.89 mV dec^−1^) and IrOx (59.85 mV dec^−1^), showing significantly improved kinetics for OER of a hierarchical nanoporous structure. The Nyquist diagrams of the EIS of NP-NF@NFF, NFF and IrOx at 1.5 V vs. RHE are shown in Figures 4D; Supplementary Figure S6. These three samples show an analogous semicircle, the equivalent circuit and fitting results shown in Supplementary Figure S7, Supplementary Table S1, showing that their charge transfer properties and electrochemical mechanism toward OER are comparable. The NP-NF@NFF shows the smallest Rct of 0.51 Ω, which is much smaller than that of NFF (2.11 Ω) and IrOx (5.7 Ω). The large specific surface area and hierarchical nanoporous structure enabled the fast mass transfer, and the enhanced conductivity accelerated the electron transfer.

**TABLE 1:** Comparison of catalyst activity in the OER.

Catalyst	$\eta_{onset}$ (mV)	$\eta_{j=10{\;mA\;cm}^{-2}}$ (mV)	$\eta_{j=100{\;mA\;cm}^{-2}}$ (mV)	Tafel slope (mV dec^-1^)
NP-NF@NFF	185	210	0.285	32.84
NFF	225	268	0.357	43.89
IrOx	235	294	0.455	59.85

In addition to activity, the stability of electrocatalysts towards OER is critical for energy conversion systems. The electrochemical stability was tested using the chronopotentiometry method and CV curves. Encouragingly, at a potential of 1.53 V vs. RHE, the NP-NF@NFF electrode produced a current density starts at 108 mA cm^−2^ and then stabilises around this value during the 60-h reaction session, with a small current attenuation of 8 mA cm^−2^ (Figure 4E). In contrast, the current densities of NFF and IrOx electrode was lower than the NP-NF@NFF electrode and gradually decayed in varying degrees (Figures S8, 9). Additionally, both NP-NF@NFF and NFF possess a much higher stability than commercial IrOx, which can be ascribed to the excellent intrinsic stability of Ni-Fe catalyst and the sturdiness of the integrated electrode. The LSV curve of NP-NF@NFF after 60 h chronopotentiometry test (NP-NF@NFF-60h) was shown in Supplementary Figure S10, which exhibits only a slight degradation of current density compared with the initial electrode, certifying the prominent stability of NP-NF@NFF. Meanwhile, as shown in Supplementary Figure S11, the LSV curve of NP-NF@NFF exhibits negligible loss of current density after testing 2500 cycles of CV also confirmed the excellent stability. These results satisfy the requirements of commercial alkaline water electrolysers (Hall, 1985). Altogether, these results show that NP-NF@NFF has an effective and stable OER performance because it has the advantages of large multiple active sites, Ov, and heterojunctions.

## 4 Conclusion

Dealloying is an excellent synthetic method for producing nanoporous structures. On the basis of the operability of dealloying, it was possible to obtain NP-NF@NFF with a hierarchical nanoporous structure from commercial NFF. Because of the uniqueness of the dealloying pore-forming technology, a porous structure is formed while endowing NP-NF@NFF with Ov and heterojunction characteristics. With its unusual nanoporous structure, NP-NF@NFF demonstrates superior OER performance over a state-of-the-art IrOx electrocatalyst, which produces an overpotential of 210 and 285 mV at 10 and 100 mA cm^−2^, respectively. Additionally, NP-NF@NFF showed remarkable stability, as the current remained basically stable over the 60-h chronopotentiometric test. This study provides a novel strategy to fabricate hierarchical nanoporous structure on metal foams and is an accessible strategy for large-scale synthesis and practical application.

## Data Availability

The original contributions presented in the study are included in the article/Supplementary Material, further inquiries can be directed to the corresponding authors.
