# Supplementary material for: Dealloying fabrication of hierarchical porous Nickel–Iron foams for efficient oxygen evolution reaction
Source: Front Chem. 2022 Nov 3;10:1047398. doi: 10.3389/fchem.2022.1047398 (PMC9669365; doi:10.3389/fchem.2022.1047398)
Supplement: Supplementary file 1 [file DataSheet1.docx]

Supplementary Material

# Supplementary Figures and Tables

## Supplementary Figures


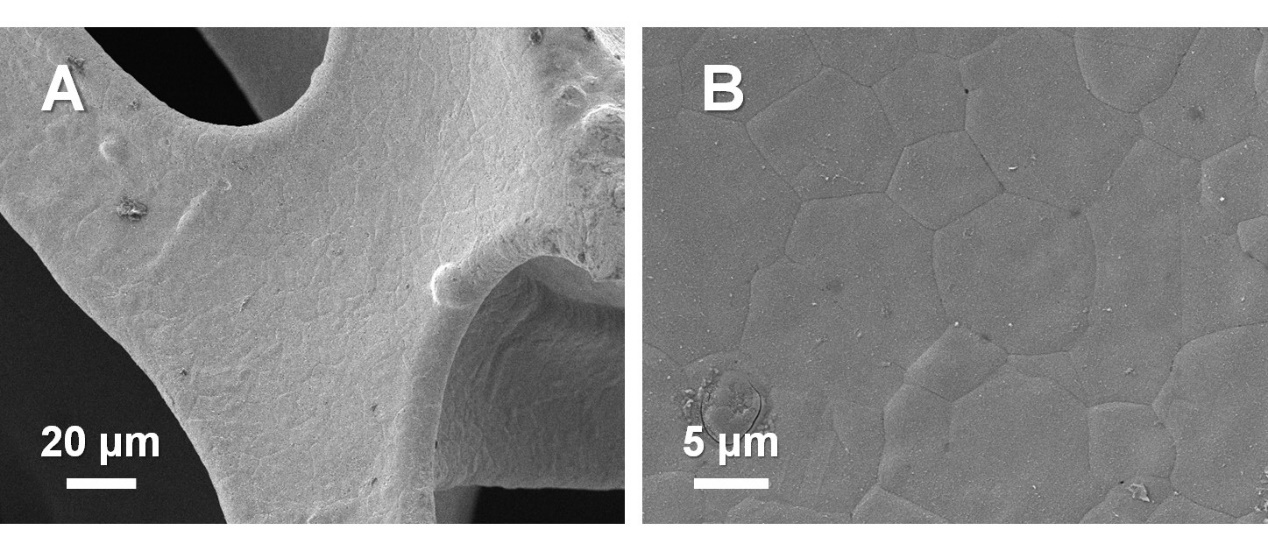


**Supplementary Figure 1.** SEM images of NFF.


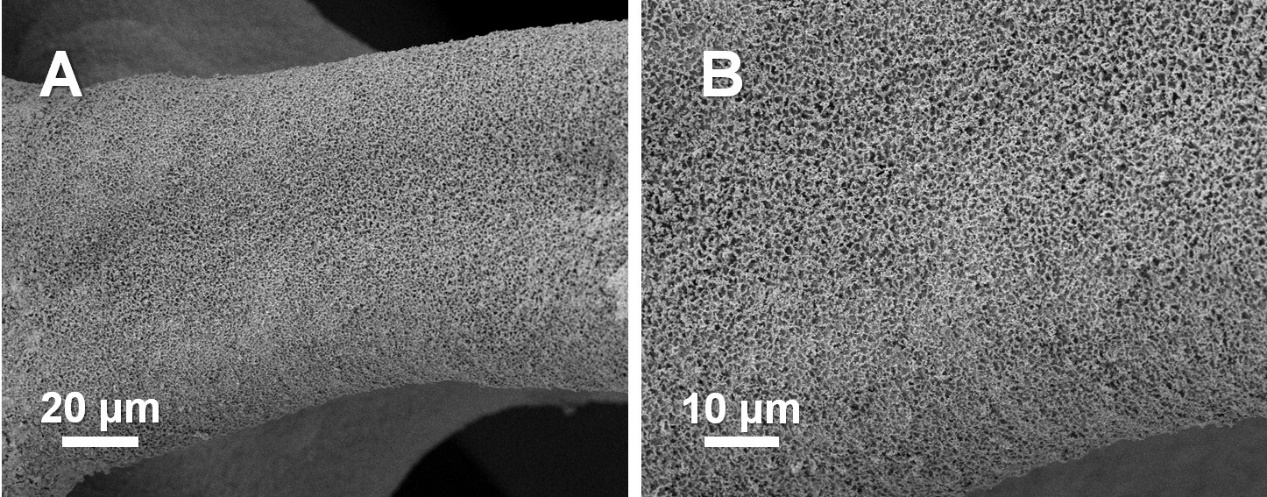


**Supplementary Figure 2.** SEM images of NP-NF@NFF.


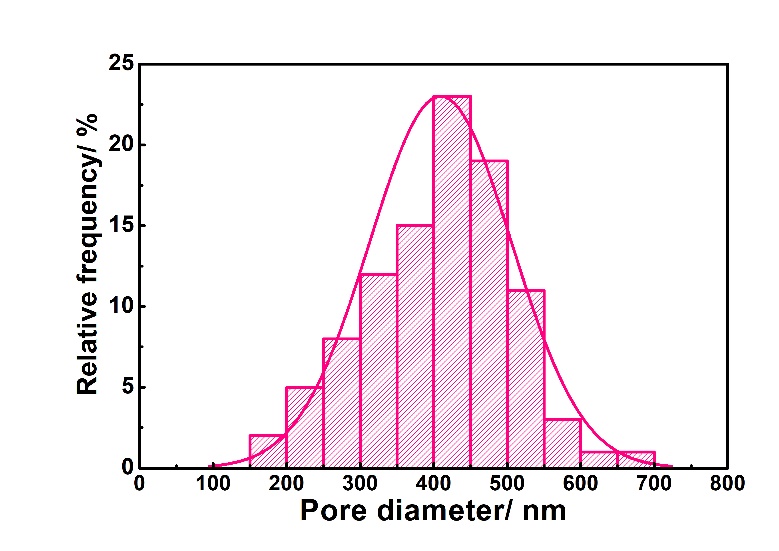


**Supplementary Figure 3.** The nanopore diameter distributions along with standard deviations of the NP-NF@NFF. The nanopore size of NP-NF@NFF is about 400 nm, which can be concluded by the Figure 1C.


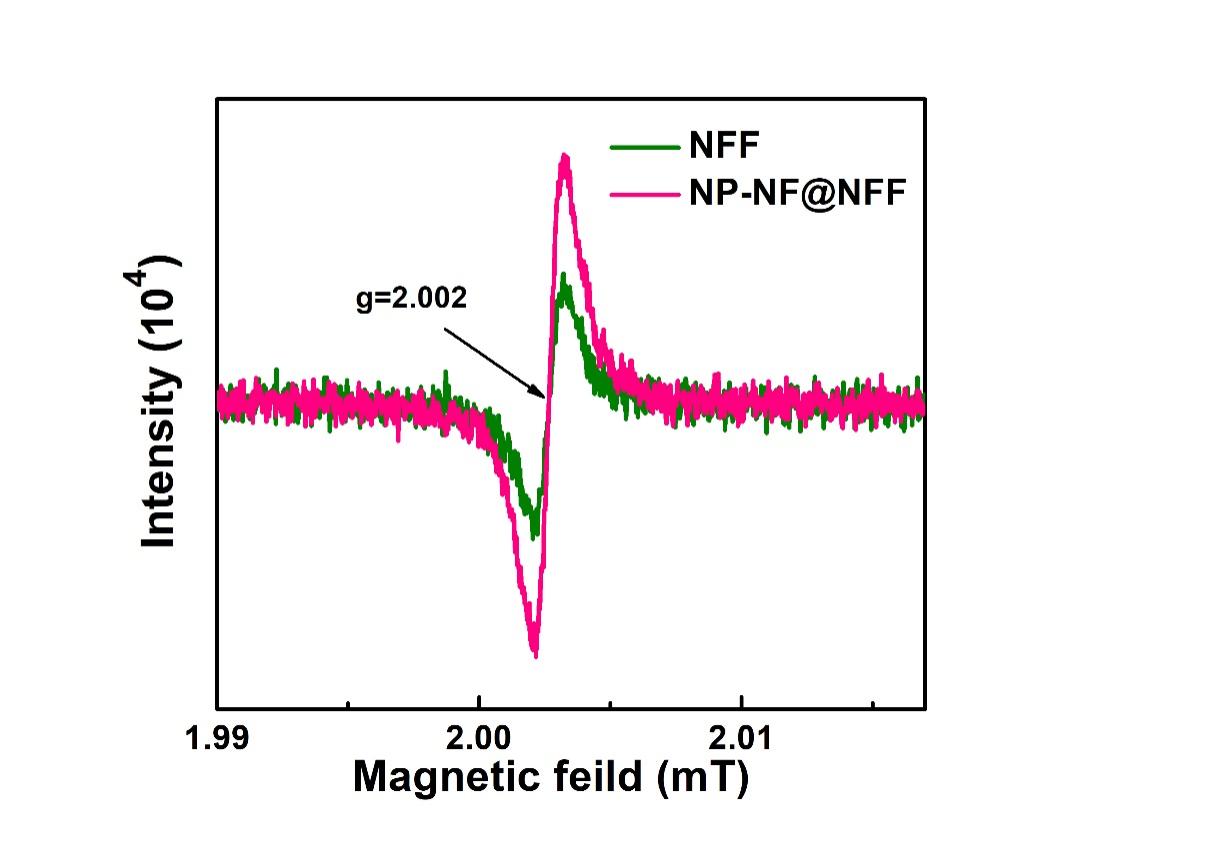


**Supplementary Figure 4.** Electron spin resonance spectra of NFF and NP-NF@NFF.


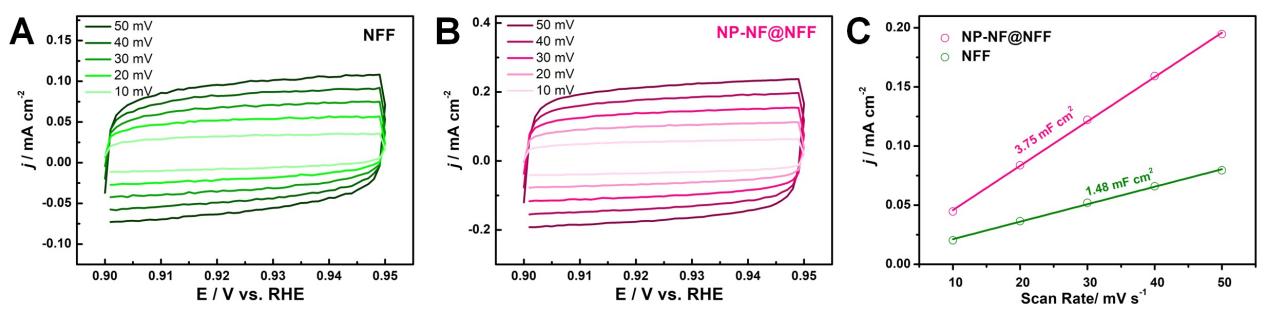


**Supplementary Figure 5.** Cyclic voltammogram curves of (a) NFF and (b) NP-NF@NFF at the different scan rates from 10 to 50 mV s**^-^**^1^ in the potential range of 0.90 – 0.95 V vs. RHE; (c) Capacitive currents at 0.925 V versus RHE as a function of scan rate for NFF and NP-NF@NFF.


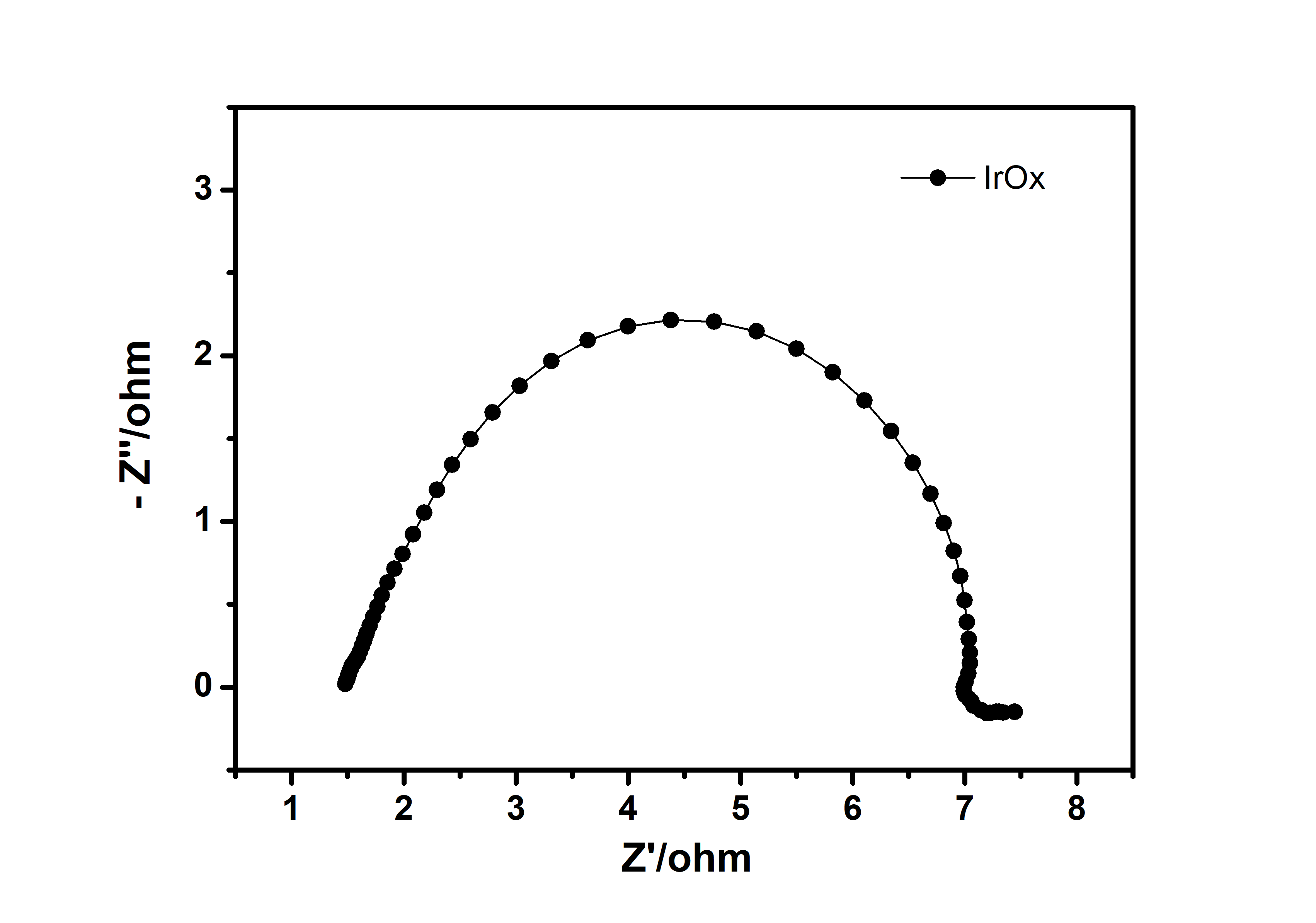


**Supplementary Figure 6.** Electrochemical impedance parameters obtained simulating the Nyquist plots of IrO*_x_*.


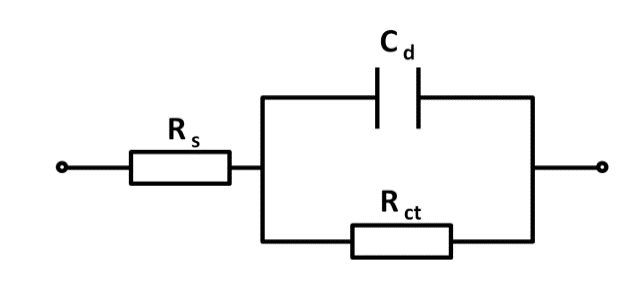


**Supplementary Figure 7.** Equivalent circuit used for fitting the EIS data.


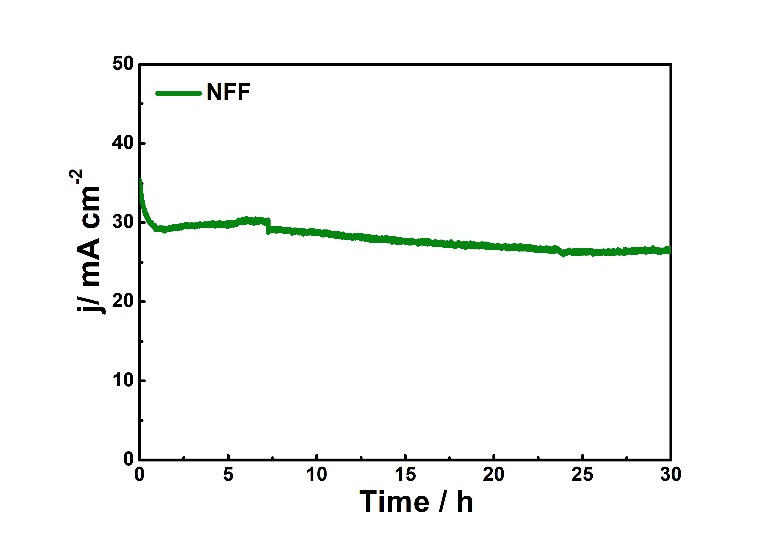


**Supplementary Figure 8.** Chronopotentiometry curve at 1.53 V vs. RHE for NFF.


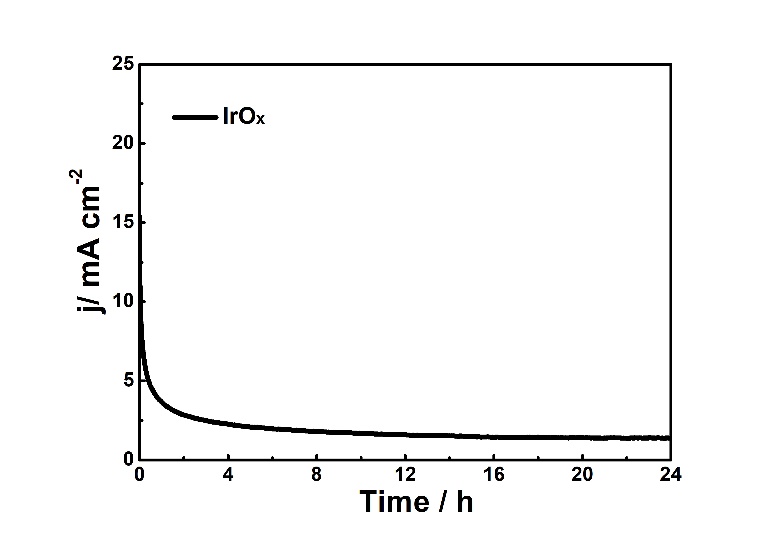


**Supplementary Figure 9.** Chronopotentiometry curve at 1.53 V vs. RHE for IrO_x_.


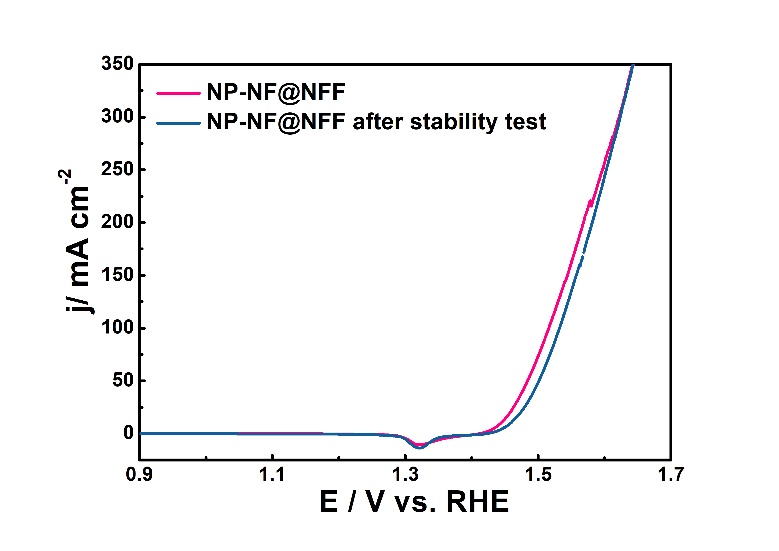


**Supplementary Figure 10.** LSV curves of the NP-NF@NFF electrode before and after 60-h I-T stability test.


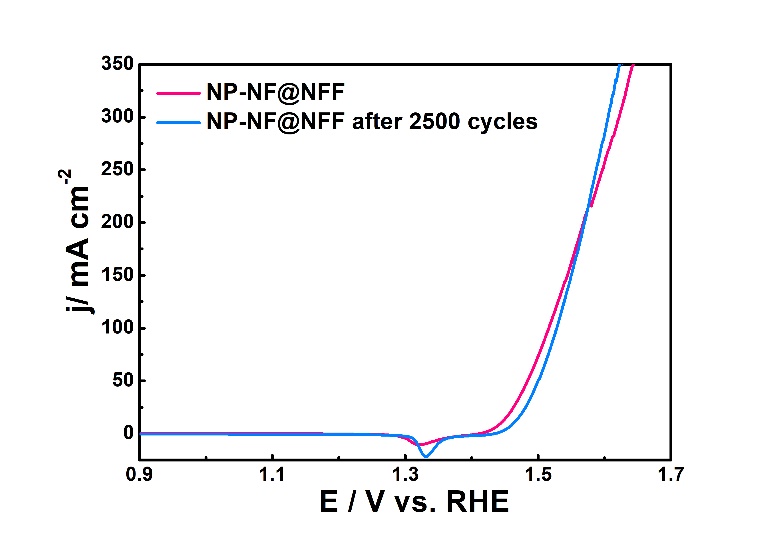


**Supplementary Figure 11.** LSV curves of the NP-NF@NFF electrode before and after 2500 cycles stability test.

## Supplementary Tables

**Supplementary Table 1.** The impedance parameters derived by fitting the EIS responses.

| **Catalyst** | **R_s_** | **Q** | **n** | **R_ct_** |
| --- | --- | --- | --- | --- |
| **NP-NF@NFF** | 1.054 | 0.1221 | 0.7625 | 0.5136 |
| **NFF** | 1.1 | 0.0197 | 0.8 | 2.166 |
| **IrO*_x_*** | 1.523 | 0.005783 | 0.8 | 5.7 |

**Supplementary Table 2.** Atomic percentage of Fe and Ni measured by XPS. The atomic proportion of Fe and Ni on the surface and interior is similar, and the proportion of Fe on the surface decreases slightly due to the influence of the dealloying process.

| Samples | Atomic % of Fe | Atomic % of Ni |
| --- | --- | --- |
| Surface of NP-NF@NFF | 43.2 | 56.7 |
| Interior of NP-NF@NFF | 39.3 | 60.7 |

**Supplementary Table 3.** Comparison of the OER activities of some recent reported NiFe-based OER electrocatalysts.

| Electrocatalysts | Electrolyte | Overotential at 10mA cm^-2^ (mV) | Overotential at 100 mA cm^-2^ (mV) | Tafel slope (mV dec^-1^) | | Ref. |
| --- | --- | --- | --- | --- | --- | --- |
| NP-NF@NFF | 1M KOH | 210 | 285 | 32.84 | This work | |
| NiFe-btz/NF-OH | 1M KOH | 239 | -- | 44.3 | (Li et al., 2022a) | |
| FeNi_3_@(Fe,Ni)S_2_-7.2 | 1M KOH | -- | 288 | 48 | (Minglei et al., 2021) | |
| NiFe MOF/Ni Foam | 1M KOH | 221 | 256 | 40 | (Liu et al., 2022a) | |
| NiFe-LDH/Co-nanocrystal | 1M KOH | 282 | -- | 64 | (Zhu et al., 2022) | |
| Anodized NiFe | 1M KOH | 346 | 500 | 56.8 | (Hashemi et al., 2022) | |
| crystalline/amorphous NiFe MOF | 1M KOH | 236 | -- | 30 | (Li et al., 2022b) | |
| NiFe CHs-CNT/G | 1M KOH | 300 | -- | 60.13 | (Liu et al., 2022b) | |
| NiFe-MOF@NF | 1M KOH | 265 | -- | 38.1 | (Xia et al., 2022) | |
| NiFe-polydopamine film | 1M KOH | 254.1 | 293 | 23.3 | (Zhang et al., 2022) | |
| Ni_2_Fe_1_ nanocages | 1M KOH | 280 | -- | 55.3 | (Wu et al., 2022) | |

**References:**

HASHEMI, N., NANDY, S., CHAE, K. H. & NAJAFPOUR, M. M. 2022. Anodization of a NiFe Foam: An Efficient and Stable Electrode for Oxygen-Evolution Reaction. *ACS Applied Energy Materials,* 5**,** 11098-11112.

LI, S., WANG, T., TANG, D., YANG, Y., TIAN, Y., CUI, F., SUN, J., JING, X., SHOLL, D. S. & ZHU, G. 2022a. Metal–Organic Framework Integrating Ionic Framework and Bimetallic Coupling Effect for Highly Efficient Oxygen Evolution Reaction. *Advanced Science,* n/a**,** 2203712.

LI, Y., MA, W., YANG, H., TIAN, Q., XU, Q. & HAN, B. 2022b. CO_2_-Assisted synthesis of a crystalline/amorphous NiFe-MOF heterostructure for high-efficiency electrocatalytic water oxidation. *Chemical Communications,* 58**,** 6833-6836.

LIU, Y., LI, X., SUN, Q., WANG, Z., HUANG, W.-H., GUO, X., FAN, Z., YE, R., ZHU, Y., CHUEH, C.-C., CHEN, C.-L. & ZHU, Z. 2022a. Freestanding 2D NiFe Metal–Organic Framework Nanosheets: Facilitating Proton Transfer via Organic Ligands for Efficient Oxygen Evolution Reaction. *Small,* 18**,** 2201076.

LIU, Y., WANG, Y., WEN, H., HAN, Y. & DENG, S. 2022b. Green Preparation of CNTs/Graphite Supported NiFe Carbonate Hydroxides for Oxygen Evolution Reaction. *ChemCatChem,* 14**,** e202200453.

MINGLEI, Y., ZHIYANG, Z., PEIXIN, C., KUN, M., CHI, C., XIZHANG, W., QIANG, W., HUI, Y., LIJUN, Y. & ZHENG, H. 2021. Construction of hierarchical FeNi_3_@(Fe, Ni)S_2_ core-shell heterojunctions for advanced oxygen evolution. *Nano Research,* 14**,** 4220-4226.

WU, J., ZHANG, W., XIAO, G., XIA, L., XIE, Q., ZHANG, C., YANG, T. & ZHAO, Y. 2022. Direct design of cage-like bimetallic NiFe hydroxides with regulated electron structure to boost the kinetic activity of oxygen evolution reaction. *Applied Surface Science,* 579**,** 152235.

XIA, L., BOWERS, C., DONG, P., YE, M. & SHEN, J. 2022. Precursor-converted formation of bimetallic–organic framework nanosheets for efficient oxygen evolution reaction. *Inorganic Chemistry Frontiers,* 9**,** 3148-3155.

ZHANG, L., TANG, W., DONG, C., ZHOU, D., XING, X., DONG, W., DING, Y., WANG, G. & WU, M. 2022. Bionic sunflower-like structure of polydopamine-confined NiFe-based quantum dots for electrocatalytic oxygen evolution reaction. *Applied Catalysis B: Environmental,* 302**,** 120833.

ZHU, S., WANG, J., LI, H., CAI, J., LI, Y., HU, J., HE, Y. & ZHOU, Y. 2022. NiFe Layered Double Hydroxide Nanosheets Anchored on Cobalt Nanocrystal Matrixes as Electrocatalysts for Oxygen Evolution. *ACS Applied Nano Materials,* 5**,** 13047-13054.
